# Pushing Boundaries: Robotic Nephrectomy of an Auto-transplanted Kidney for Recurrent Renal Cell Carcinoma

**DOI:** 10.7759/cureus.2280

**Published:** 2018-03-06

**Authors:** Belinda Li, Eric J Kirshenbaum, Parth Patel, Alex Gorbonos

**Affiliations:** 1 Urology, Loyola University Chicago, Stritch School of Medicine, Maywood, Illinois

**Keywords:** renal cell carcinoma, kidney transplant, autotransplant, robotic surgery

## Abstract

Advances in robotic technology continue to expand the boundaries of minimally invasive approaches in transplant surgery. A single report has previously described the use of the robotic approach in transplant nephrectomy for a failed allograft. Our objective is to describe our technique and experience for the first reported robotic nephrectomy of an auto-transplanted solitary kidney for a recurrence of renal cell carcinoma (RCC). We highlight technical considerations during allograft mobilization and hilum dissection with the additional demands of a previously operated auto-transplant kidney.

## Introduction

Renal auto-transplantation is a complex procedure which has been described for select cases of ureteral injuries, malignancies, renal vascular pathology, retroperitoneal fibrosis, and loin pain-hematuria syndrome [1]. Due to the inherent decreased risk of auto-transplant rejection, there is a lack of literature describing the subsequent reoperation or explant for these kidneys. However, several case reports and series exist on conventional renal transplant nephrectomies, most commonly in the setting of a failed renal allograft due to acute or chronic rejection [2-3].

The classic open surgical approach to transplant nephrectomy is through the prior Gibson incision, either intracapsular or extracapsular [2]. Interval regional scarring and fibrosis result in a technically difficult and risky procedure, contributing to a high rate of serious complications (up to 21.4%) including bleeding and wound infections [3].

The advent of minimally invasive surgery has revolutionized patient-related postsurgical outcomes, including reduced pain, shortened length of stay, and improved cosmesis. While minimally invasive surgery has become the standard for numerous intra-abdominal and retroperitoneal procedures including partial and radical nephrectomy, there is only one published report of a robotic approach to transplant nephrectomy for a failed renal allograft [4]. To our knowledge, this approach has not been reported for an auto-transplant kidney. We report the first robotic-assisted laparoscopic nephrectomy of an auto-transplant kidney, performed for tumor recurrence in a solitary kidney that had previously undergone ex-vivo partial resection, for a complex renal cell carcinoma (RCC).

## Case presentation

Case report

The patient is a 64-year-old man who previously underwent a right radical nephrectomy for RCC. Four years following his nephrectomy, he presented with a new centrally located left renal mass measuring 5.3 cm. He declined radical nephrectomy to avoid dialysis and underwent a nephron-sparing approach with a laparoscopic nephrectomy, ex-vivo partial nephrectomy and reconstruction, and auto-transplant to the right iliac fossa. Pathology confirmed a clear cell RCC with negative surgical margins.

One year later, he was found to have a recurrence near the renal pelvis measuring approximately 3 cm. Again, the patient declined radical nephrectomy and was thus started on tyrosine kinase inhibitor (TKI) therapy; serum creatinine was 3.4 mg/dl at that time. The mass remained stable on TKI therapy for four years until the medication was discontinued due to toxicity with recurrent Clostridium difficile colitis.

On follow-up imaging one year thereafter while off systemic therapy, the mass had increased in size from 3 cm to 6 cm. In the interval, he had also begun hemodialysis for progressive renal deterioration and end-stage renal disease. At that time, he was referred for consideration of transplant auto-nephrectomy. He was counseled on open versus minimally invasive techniques and elected for a robotic approach.

Description of technique

**Figure 1:**
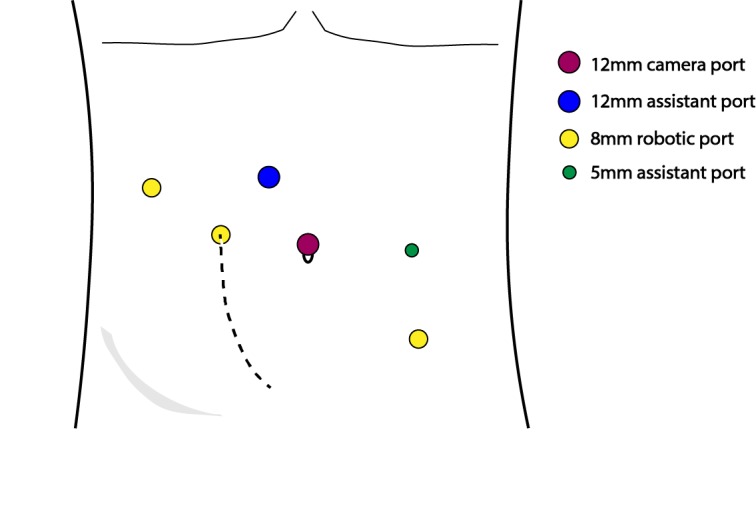
Port placement (dotted line represents patient’s prior Gibson incision)

The procedure was performed transperitoneally using the da Vinci Si Surgical System. The patient was positioned supine in slight Trendelenburg and prepped and draped in standard fashion. The robot was docked from the patient’s right side. A 12mm camera port was placed at the umbilicus and three robotic ports were utilized in addition to two assistant ports and placed as shown in Figure 1.

**Figure 2:**
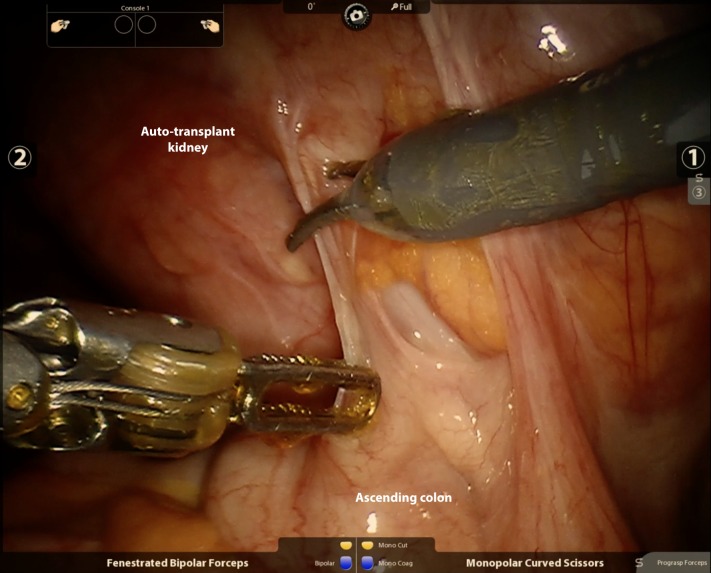
Mobilization of ascending colon

**Figure 3:**
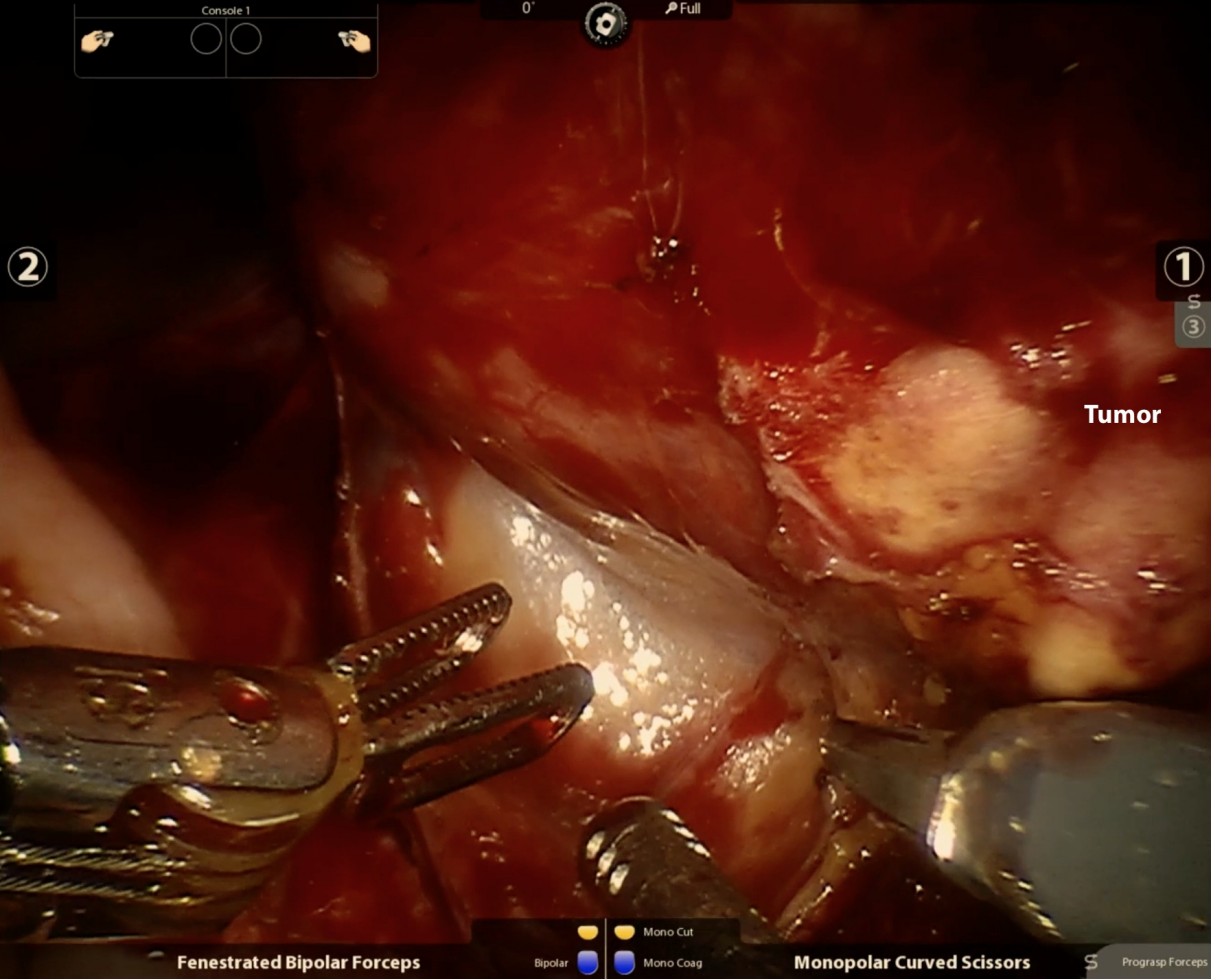
Visualization of tumor

Once the robot was docked, the notably adherent ascending colon was first mobilized off of the transplant kidney (Figure 2). Monopolar scissors and fenestrated bipolar forceps were used for the majority of the initial dissection. The fourth robot arm assisted with retraction using ProGrasp^TM^ forceps (Intuitive Surgical, Sunnyvale, CA). Once adhesions were released, the contour of the tumor was readily seen (Figure 3). A challenging and meticulous dissection was then performed to completely mobilize the kidney circumferentially around the hilum, initially obscured due to adhesions. Eventually, the kidney was freed sufficiently to reveal the vascular pedicle to the right external iliac vessels as we prepared to divide this with the Endo GIA^TM^ stapler (Intuitive Surgical, Sunnyvale, CA). The transperitoneal approach offers excellent visualization of the common iliac artery after mobilization of the kidney, which can be held in place with the assistance of the fourth arm.

**Figure 4:**
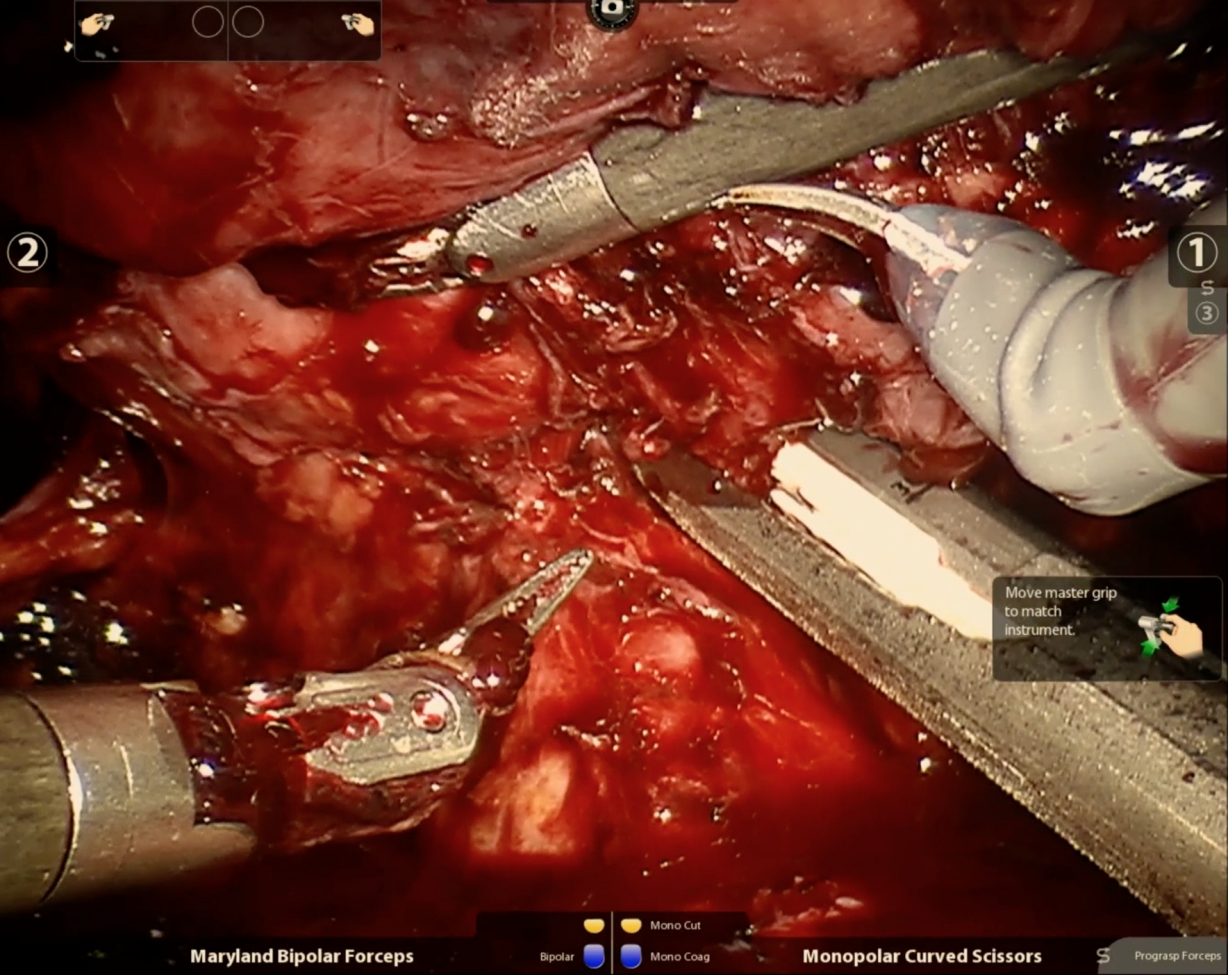
Test clamp across the renal artery prior to deploying the laparoscopic stapler Lower extremity pulses should be palpated to ensure absence of vascular compromise.

The renal artery was divided first, but here we emphasize the importance of palpating the right lower extremity pulses to ensure that vascular supply to the lower extremity is not compromised. The shortened renal artery length and its angulation pose a risk for clamping of the iliac arteries. Test clamping should be performed with the stapler prior to deploying the instrument (Figure 4). The renal vein and ureter were then divided sequentially in a similar fashion.

**Figure 5:**
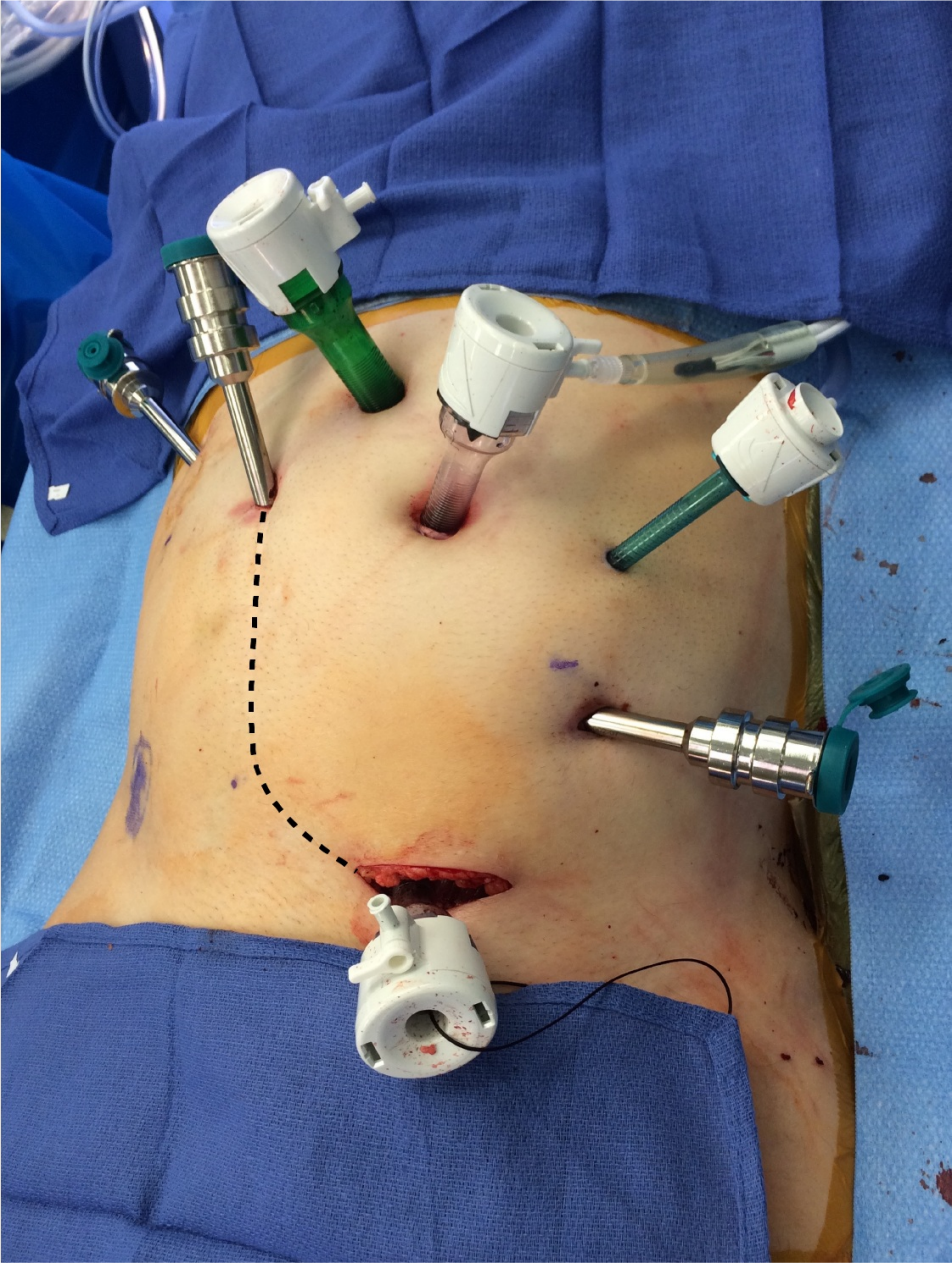
Specimen extraction site at the lower medial aspect of the patient’s prior Gibson scar

To prepare for specimen extraction, a smaller incision was extended from the medial aspect of the patient’s previous Gibson incision (Figure 5).

## Discussion

Results

The duration of the case was 387 minutes. Estimated blood loss (EBL) was 800 mL. The patient received two units of packed red blood cells intraoperatively and none postoperatively. He was admitted to a surgical floor and started immediately on a clear liquid diet to be advanced as tolerated. Early ambulation was encouraged. Analgesia was provided with scheduled acetaminophen and oral oxycodone as needed. Intravenous narcotics were available but not requested. The patient continued to receive hemodialysis according to his preoperative schedule. He was discharged on postoperative day three after an unremarkable course. His final pathology revealed a recurrent clear cell RCC measuring 8.2 cm, Fuhrman grade 4, with renal vein involvement.

Discussion

The reported incidence of transplant nephrectomy is between 0.5%-43.5% [5]. Indications for transplant nephrectomy include acute rejection, chronic rejection, infection, gross hematuria, renal vein thrombosis, renal artery thrombosis, malignancy, and graft rupture [2]. Robotic transplant nephrectomy has previously been shown to be safe and feasible in the management of a failed allograft in a single report [4]. However, to our knowledge, robotic auto-transplant nephrectomy for a malignancy has not been reported. We demonstrate that the robotic approach is technically feasible for this challenging operation, which has historically been associated with significant patient morbidity.

Malignancy is a rare indication for transplant nephrectomy [3]. In fact, most de novo RCC in transplant recipients occur in the native kidneys rather than the allograft. Over time, urologists and transplant surgeons are likely to face an increasing number of allograft RCC cases due to longer graft survival from improvements in immunosuppression, transplant techniques, and patient selection. Currently, no guidelines exist on the treatment of this rare occurrence. Traditionally, open allograft nephrectomy was considered routine due to concerns about difficulty with parenchymal dissection and hilar control in a preoperative field and a fear of progressive disease in an immunosuppressed patient. It is critical to continue to improve our surgical techniques to reduce the morbidity and mortality associated with this procedure. Our described case of robotic auto-transplant nephrectomy shares important surgical principles with those of a failed allograft nephrectomy with the additional technical considerations of significant neovascularization and avoiding tumor violation.

Historically in open surgery, the most common complication of the procedure was wound infections due to a combination of factors, including immunosuppression and size of incisions [5]. In addition, the rate of major postoperative hemorrhage has been reported to be 15% with high associated mortality. As surgical experience has grown, mortality in more recent years have decreased but remain problematic as seen in Bonilla’s [3] recent series with mortality rates of 1.9% and 1.1%, respectively. Other reported complications include ligation of iliac vessels, lymphocele, urinary fistula, obturator nerve injury, and bowel injury.

Our initial experience using robotic-assisted surgery in this unusual case of a renal auto-transplant nephrectomy showed comparable blood loss with a shorter length of stay compared to open series [3,5]. While shown to be technically feasible, there are limitations to this approach which warrant discussion. Robotic transplant or auto-transplant nephrectomy is a specialized procedure which requires a trained staff with a depth of experience in robotic surgery. Laparoscopic staplers rely on skilled bedside assistants. The newer EndoWrist® (Intuitive Surgical Inc., Sunnyvale, CA) robotic stapler has not yet been reported for use during this procedure. A robotic approach does require entering the peritoneal cavity which would not be true of an open extraperitoneal operation, placing the patient at risk for bowel complications or ileus. There are limitations in the options for vascular ligation due to a shorter pedicle compared to native nephrectomies. Additionally, proximity to the iliac vessels carries a potentially catastrophic risk of hemorrhage in a small working space. Our operative time for this index case was longer at 387 minutes compared to those reported in open series (up to 210) [5] and to that reported in Mulloy et al.’s robotic allograft nephrectomy at 235 minutes [4], likely accounted for by the extensive additional scarring related to our patient’s prior partial nephrectomy and auto-transplant. Additional time in this case was also dedicated to ruling out a colon injury during mobilization of the cecum, which was carefully inspected and determined to be intact. The patient suffered no adverse bowel complications postoperatively. Our account hopes to further the boundaries of robotic surgery where more experience will continue to improve patient outcomes.

## Conclusions

Robotic-assisted laparoscopic auto-transplant nephrectomy is a technically challenging but feasible procedure. A minimally invasive approach can significantly reduce hospital stay and recovery period while maintaining similar operative times and blood loss compared to open alternatives. Expansion of robotic programs and continued experience will hope to further implement minimally invasive approaches for similarly complex pathologies.
